# Growth of Fe_3_O_4_ Truncated Cubes and Rhombic Dodecahedra Showing Interior Lattice and Magnetic Behavior Variations

**DOI:** 10.1021/acs.inorgchem.5c00442

**Published:** 2025-04-23

**Authors:** Jou-Hsin Yang, Chia-Peng Wang, Bo-Hao Chen, Michael H. Huang

**Affiliations:** † Department of Chemistry, National Tsing Hua University, Hsinchu 300044, Taiwan; ‡ National Synchrotron Radiation Research Center, Hsinchu 300092, Taiwan

## Abstract

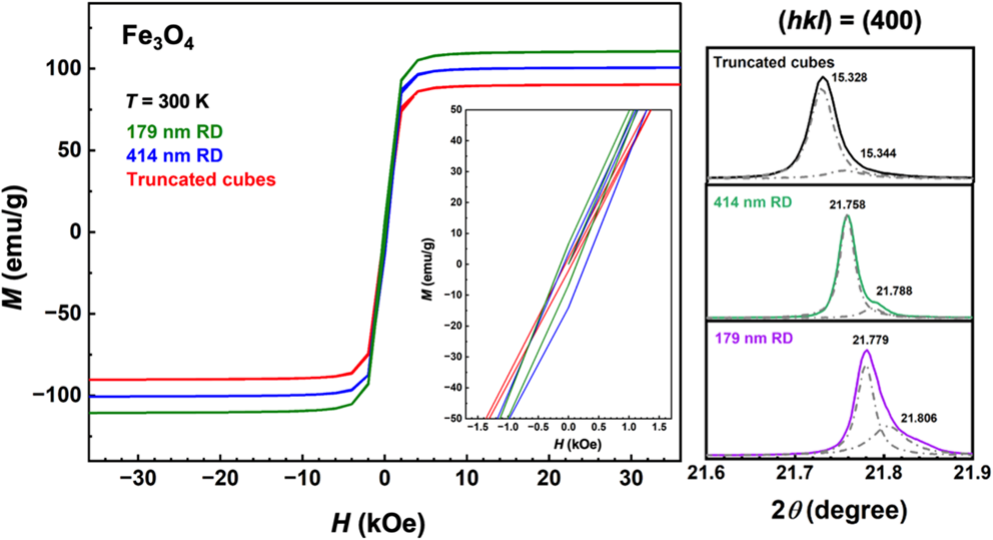

Fe_3_O_4_ edge-truncated cubes, corner-truncated rhombic dodecahedra, and rhombic dodecahedra with three different sizes were solvothermally synthesized. Besides in-house X-ray diffraction (XRD) compositional confirmation, synchrotron XRD patterns reveal the presence of bulk and surface layer lattices for all the samples, with the 179 nm rhombic dodecahedra having a thick surface layer. Truncated cubes and 179 nm rhombic dodecahedra show a cell constant difference of 0.18%. High-resolution transmission electron microscopy (HR-TEM) analysis manifests significant lattice spot deviations in the surface layer region. These crystals present size- and surface-dependent band gaps. While all the measured samples have large saturation magnetization numbers, the 179 nm rhombic dodecahedra have the largest saturation and remanent magnetization and coercivity values at both 5 and 300 K, followed by the 414 nm rhombic dodecahedra and truncated cubes. Both the optical and magnetic behaviors can be understood to arise from their interior lattice variations, which should be applicable to other magnetic ionic crystals.

## Abstract 2

Fe_3_O_4_ polyhedral crystals show size- and shape-related optical and magnetic behaviors. Interior lattice differences should explain the emergence of these phenomena.

## Introduction

Semiconductor crystals or ionic solids show facet-dependent behaviors in all kinds of properties including electrical conductivity, photocatalytic activity, light absorption and emission, magnetism, piezoelectricity, ferroelectricity, and pyroelectricity.^1−12^ Presumably, superconductivity should also be shape-related. The emergence of all these phenomena likely comes from lattice variations between the interior bulk and the surface layer inside a crystal, and the surface layer lattice can be facet-specific.^9,13^ Moreover, different particle shapes and sizes can exhibit some lattice constant changes.^11^ These lattice features appear naturally when the crystals are synthesized, as required by thermodynamics. Crystal lattices are just like different parts of a tuna, which can have dissimilar meat colors and textures. Recent synchrotron X-ray diffraction (XRD) analysis and processing of HR-TEM images reveal these structural facts.^9,11,13^ Since this is a new and real structural model of ionic solids, it is important to demonstrate the existence of this crystal structure in other materials. Previously, antiferromagnetic MnS and Co_3_O_4_ cubes and octahedra show notable temperature-related magnetization responses.^8,11^ Co_3_O_4_ octahedra also display much higher field-dependent magnetization values than cubes do. Formation of magnetite, or Fe^2+^Fe_2_^3+^O_4_, with well-defined particle shapes should be interesting to further explore how the crystal lattices tune their optical and magnetic properties.^14,15^ Fe_3_O_4_ magnetite possesses a cubic inverse spinel structure. Its unit cell contains 56 atoms with 32 oxygen atoms, 16 Fe^2+^ ions, and 8 Fe^3+^ ions. Fe^2+^ ions occupy octahedral positions, while Fe^3+^ ions take up both the octahedral and tetrahedral sites. The exchange of a spin-down d orbital electron between neighboring octahedral Fe^2+^ and Fe^3+^ ions leads to annihilation of spin moments of Fe^3+^ ions.^16,17^ Ferrimagnetism results from the unpaired spins of Fe^2+^ ions in the octahedral sites.

Fe_3_O_4_ octahedral and rhombic dodecahedral microcrystals have been synthesized hydrothermally by preparing an aqueous mixture of FeSO_4_, polyvinylpyrrolidone, hydrazine, and NaOH.^18^ Octahedral Fe_3_O_4_ microcrystals were also produced in ethylene glycol.^19,20^ For optical characterization, smaller particles are more desirable. Much smaller Fe_3_O_4_ octahedra, truncated octahedra, and rhombic dodecahedra have been synthesized by adding oleic acid and olyelamine in benzyl ether and heated to around 300 °C for 30 min.^21^ However, their optical and magnetic properties have not been reported. Magnetite formation with shape evolution from cubic to octahedral structures was successful by heating Fe(acac)_3_, oleic acid, cetylpyridinium chloride, and benzyl ether to 280 °C for 15 min.^22^ Shape-related magnetic behaviors are clearly present. Moreover, the blocking temperature (*T*_B_) also varies considerably with particle shape, above which thermal fluctuations induce random flipping of the magnetic moment. Nanoparticles then lose their stable magnetic order and become superparamagnetic.^23^ Surface coordination symmetry and interaction with adsorbed molecules have been proposed to reduce surface anisotropy.^20,22^ Consequently, different particle shapes can exhibit distinct magnetic behaviors. Calculations have also shown different distributions of surface-disordered spins in Zn_0.4_Fe_2.6_O_4_ cubes and spheres.^24^ However, possible surface spin disorientation should not produce over 10-fold magnetization difference between Co_3_O_4_ cubes and octahedra.^8^ One or two atomic layers also cannot cause observable color changes to the crystals, so this idea fails to account for the optical facet effect. More convincingly, shape-related interior lattice variations have been demonstrated; therefore, one can no longer assume a perfect ionic crystal interior for all particle shapes. Instead, a high-resolution crystal lattice examination is required to understand various facet-dependent properties including magnetic responses, just like green and yellow kiwi fruit differing beyond their surface appearance.

In this study, Fe_3_O_4_ edge-truncated cubes, corner-truncated rhombic dodecahedra, and size-tunable rhombic dodecahedra were obtained through solvothermal synthesis. Synchrotron XRD patterns reveal size- and shape-related peak shifts, as well as the presence of two lattice components in each sample. FFT-processed HR-TEM images show greater lattice point deviations in the surface region. The crystals have size- and shape-associated band gaps. Temperature- and field-varying magnetization measurements suggest that the interior crystal lattice variations should contribute to their magnetic behaviors.

## Results and Discussion

### Synthesis and XRD Characterization of Fe_3_O_4_ Polyhedra

A solvothermal method was adopted to synthesize Fe_3_O_4_ polyhedra. The source of iron ions is FeSO_4_. LiNO_3_ serves as a mild oxidant to convert ferrous ions to ferric ions. Hydrogen peroxide is a stronger oxidant and is a source of oxygen through its decomposition. After the addition of urea to the reaction solution, the solution pH remained neutral. However, after heating in an oven, the solution pH value with the final product became basic. Urea acts as a source of hydroxide ions. Fe_3_O_4_ nanocrystals prepared in organic solvents are able to achieve better crystallinity and produce monodisperse nanoparticles.^25^ For the synthesis of edge-truncated cubes and corner-truncated rhombic dodecahedra, 1-butanol was chosen after optimization testing. Toluene was selected to prepare size-tunable rhombic dodecahedra. Oxidant concentration, heating time, and temperature must be balanced to avoid overoxidation and achieve high shape control. The following reactions can occur to generate Fe_3_O_4_.
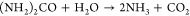
1
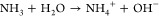
2
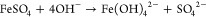
3
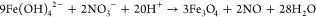
4
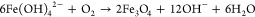
5

Particle shape control was achieved by varying the amount of the oxidant. This variation changes the reaction quotient *Q* and the cell potential *E*. Consequently, Δ*G* is changed. Assuming that Δ*H* does not really differ in forming different particle shapes, Δ*G* should mainly come from Δ*S*. This suggests that the interior lattice cannot be identical, causing the observed semiconductor facet effects. Of course, slight lattice variation can also occur in particles of different sizes. It is like sashimi prepared by a skilled chef can offer a different tasting experience, even though it starts with the same fish.

**Figure 1:**
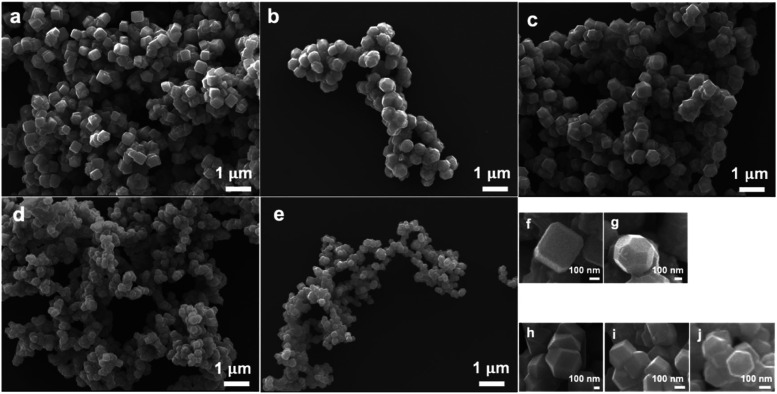
SEM images of the as-synthesized Fe_3_O_4_ (a) truncated cubes, (b) truncated rhombic dodecahedra, (c) 414 nm rhombic dodecahedra, (d) 266 nm rhombic dodecahedra, and (e) 179 nm rhombic dodecahedra. Enlarged SEM images of (f, g) truncated cubes and truncated rhombic dodecahedra and (h–j) size-tunable rhombic dodecahedra.

**Figure 2:**
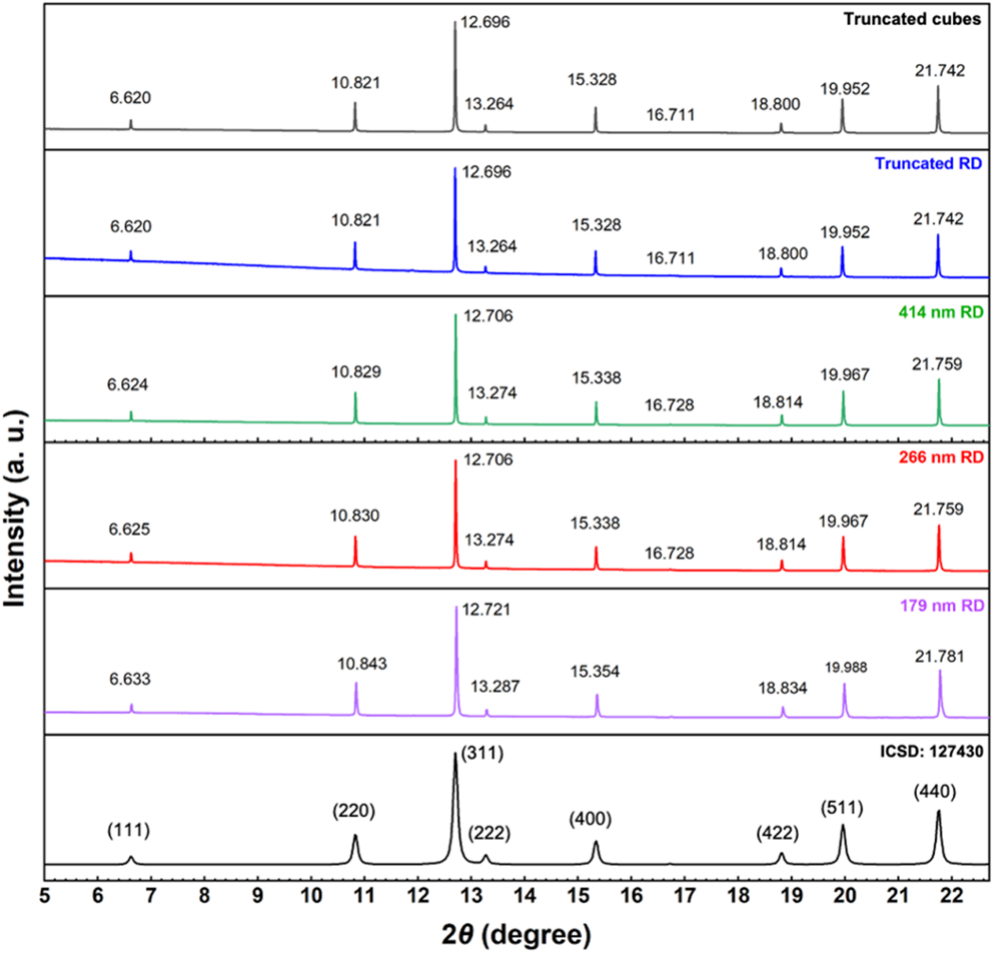
Synchrotron XRD patterns of the synthesized Fe_3_O_4_ crystals.

**Figure 3:**
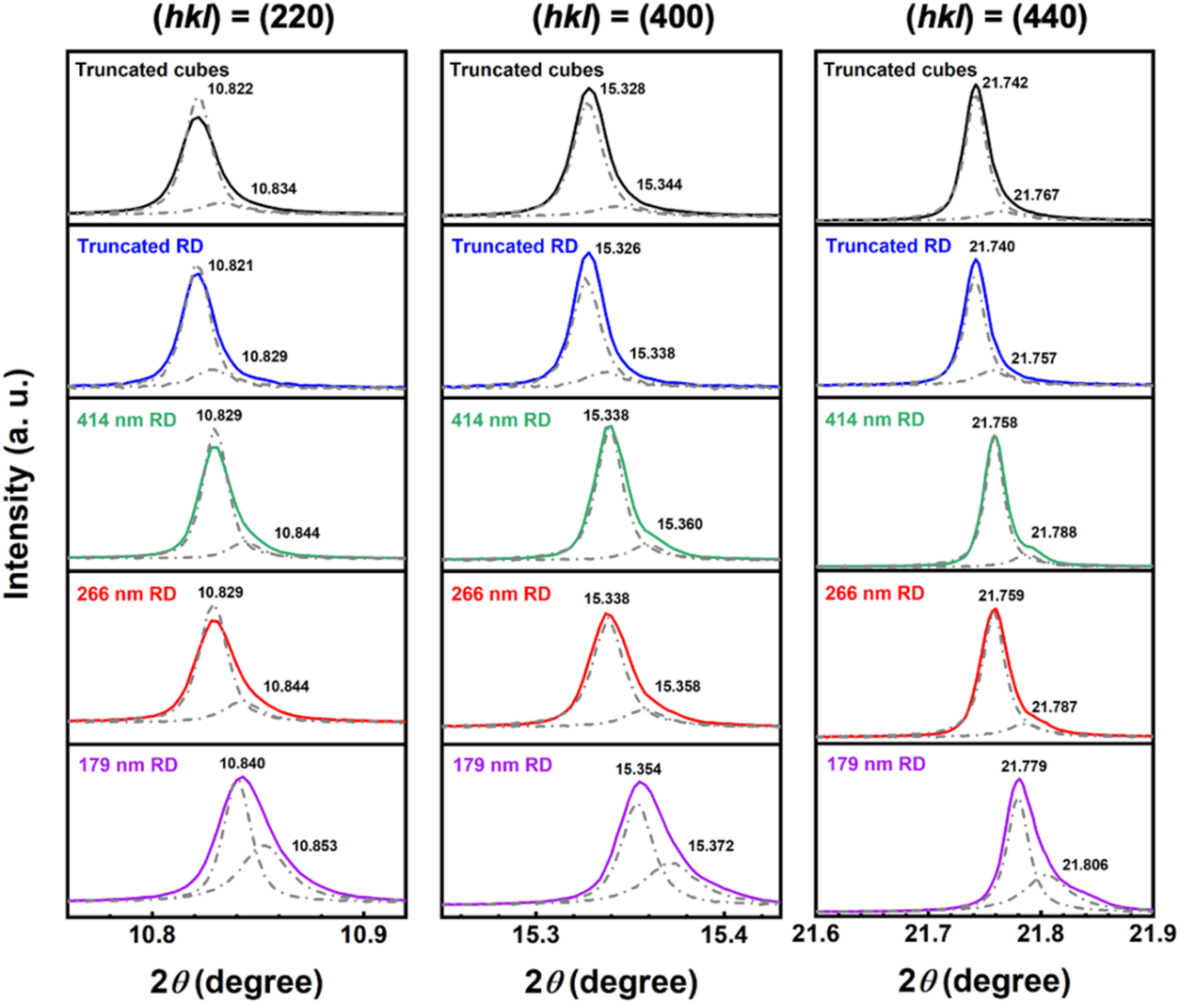
Selected XRD peaks of Fe_3_O_4_ crystals with Rietveld refinement, revealing two lattice components.

Figure 1 presents scanning electron microscopy (SEM) images of the synthesized Fe_3_O_4_ edge-truncated cubes with an average edge length of 348 nm, corner-truncated rhombic dodecahedra with an average size of 460 nm and 414, 266, and 179 nm rhombic dodecahedra. The edge-truncated cubes and corner-truncated rhombic dodecahedra differ in the proportions of the {100} and {110} faces. Their size distribution histograms are provided in Figure S1. The particles have high size and shape uniformity. Figure S2 provides the in-house XRD patterns of these samples. While all of the samples indicate exclusive formation of magnetite, shape- and size-related peak shifts are less conclusive. The preferred orientation effect is not obvious, possibly from some degree of magnetism-induced particle aggregation to prevent the formation of a monolayer of particles on the substrate. Maghemite (γ-Fe_2_O_3_) has (210) and (211) peaks, in addition to large peak shifts relative to those of magnetite, so it is easy to exclude its presence.^26^ Synchrotron XRD patterns were collected (Figure 2). After correction for peak shifts caused by human and instrument errors such as variations in sample placement, peak positions are accurate. Rietveld refinement was also conducted to determine the exact cell parameters (Figure S3 and Table S1). Truncated cubes and truncated rhombic dodecahedra have nearly identical cell constants. Relative to these samples, 414 and 266 nm rhombic dodecahedra show peaks shifting to slightly higher angles. Figure 3 reveals that the 179 nm rhombic dodecahedra further shift the peaks to higher 2θ angles. Thus, by expanding a single diffraction peak, large peak shifts can be easily recognized. Therefore, the cell constant *a* can vary with particle shape and size.

Particularly for the smallest rhombic dodecahedra, peak asymmetry means that each diffraction peak has two components (Figure 3). Other than truncated rhombic dodecahedra, a uniaxial strain model was applied to the XRD patterns. For rhombic dodecahedra, (110) crystal planes are specifically chosen to perform precise refinement calculations, while (100) planes are utilized for truncated cubes. This approach allowed us to determine equatorial and axial strains (Table S1). For rhombic dodecahedra, two different strain models were employed: for phase one, a uniaxial mode was used with the unique axis [*hkl*] being the (110) plane; for phase two, an isotropic mode was applied. The two components represent the interior bulk and the surface layer. They are distinguished by the equatorial and axial microstrains. The surface component has a larger microstrain due to stronger tensile stress. For the (220) peak, the refined peak intensity is higher than the experimental one. This is because the refinement was performed over the full profile pattern, so some selected parameters may not perfectly match all the measured peaks.

**Figure 4:**
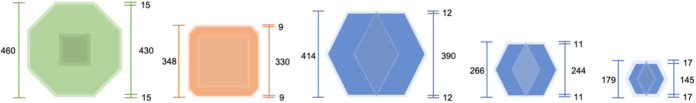
Synchrotron XRD-determined surface layer thicknesses.

The truncated cubes and truncated rhombic dodecahedra have a unit cell *a* of 8.402 Å for their crystal bulk, while that of 179 nm rhombic dodecahedra is 8.387 Å. For the surface layer, the truncated cubes and 179 rhombic dodecahedra have respective cell constants of 8.393 and 8.378 Å. That is a 0.18% change in the unit cell length. Although this number may seem quite small, an enormous cell constant change of 0.69% has been measured between SrTiO_3_ cubes and truncated rhombic dodecahedra.^27^ From the calculated particle weight percentages, the bulk component accounts for the majority of the particle weight. However, the surface layer volume approaches that of the bulk for 179 nm rhombic dodecahedra. From the determined surface layer volumes, Figure 4 gives the surface layer thicknesses. The smallest rhombic dodecahedra have a significant surface layer. This examination demonstrates again that nanoscale ionic crystals generally exhibit bulk and surface region lattices.

### TEM and X-ray Photoelectron Spectroscopic (XPS) Characterization

**Figure 5:**
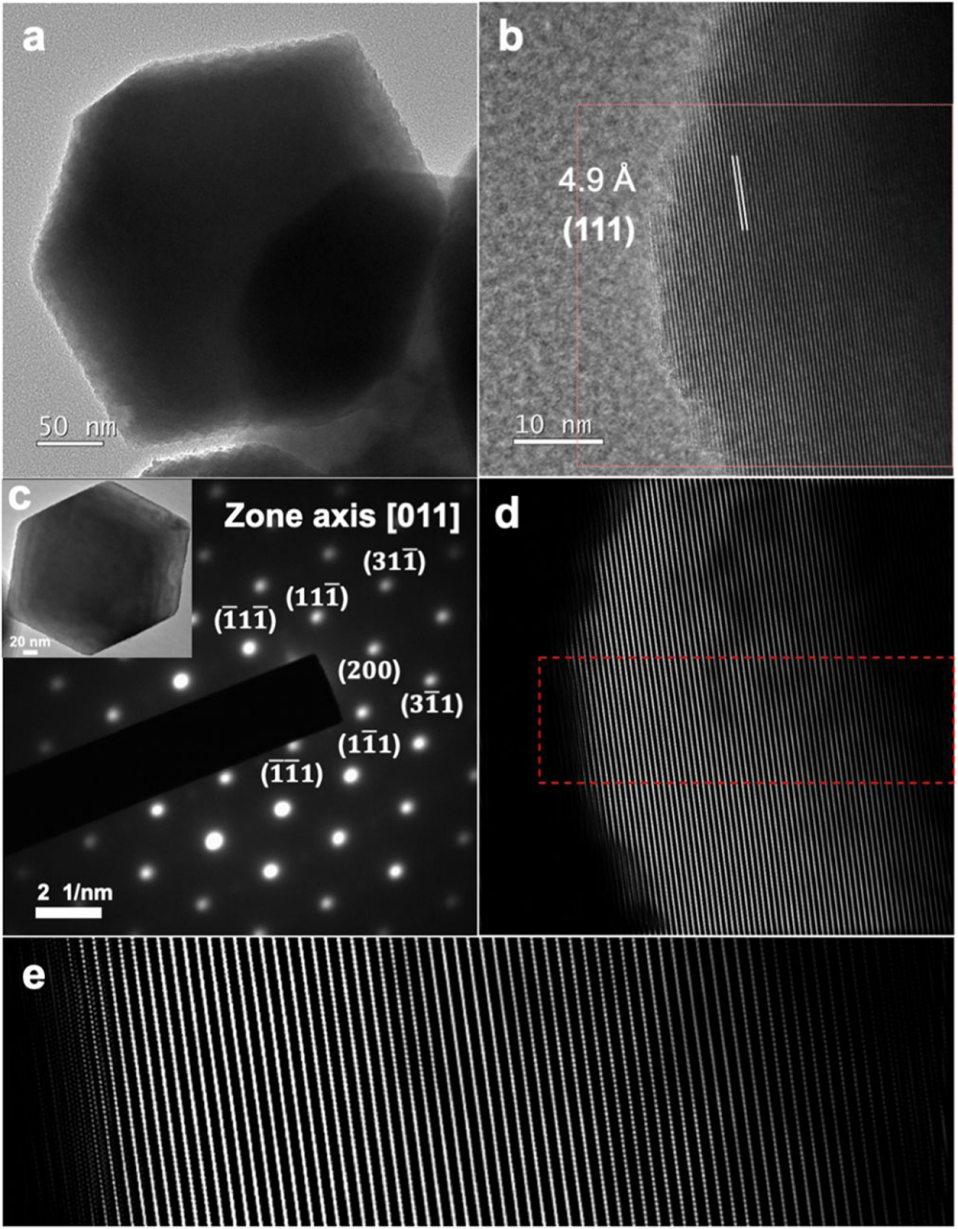
(a, b) TEM image of a 179 nm Fe_3_O_4_ rhombic dodecahedron and HR-TEM image of the left portion of the particle. (c) SAED pattern and the viewed particle TEM image. (d) FFT-processed lattice image of the red square region in panel b. (e) Zoom-in image of the dashed region.

TEM characterization was performed by using the smallest 179 nm rhombic dodecahedra (Figure 5). The recorded selected-area electron diffraction (SAED) pattern indicates single crystallinity of the particle. Figure 5b shows the HR-TEM image of a portion of a rhombic dodecahedron viewed along the [011] zone axis. Lattice fringes with a measured *d*-spacing of 4.9 Å correspond to its (111) planes. The fast Fourier transform (FFT)-processed lattice image (Figure 5d, e) shows the presence of elongated lattice points near the nanoparticle surface, where dots become connected lines. Away from the surface, the inner lattice points are discernably separated. The observation supports synchrotron XRD results of distinct lattice structures between the bulk and surface layer of the Fe_3_O_4_ nanocrystals.

**Figure 6:**
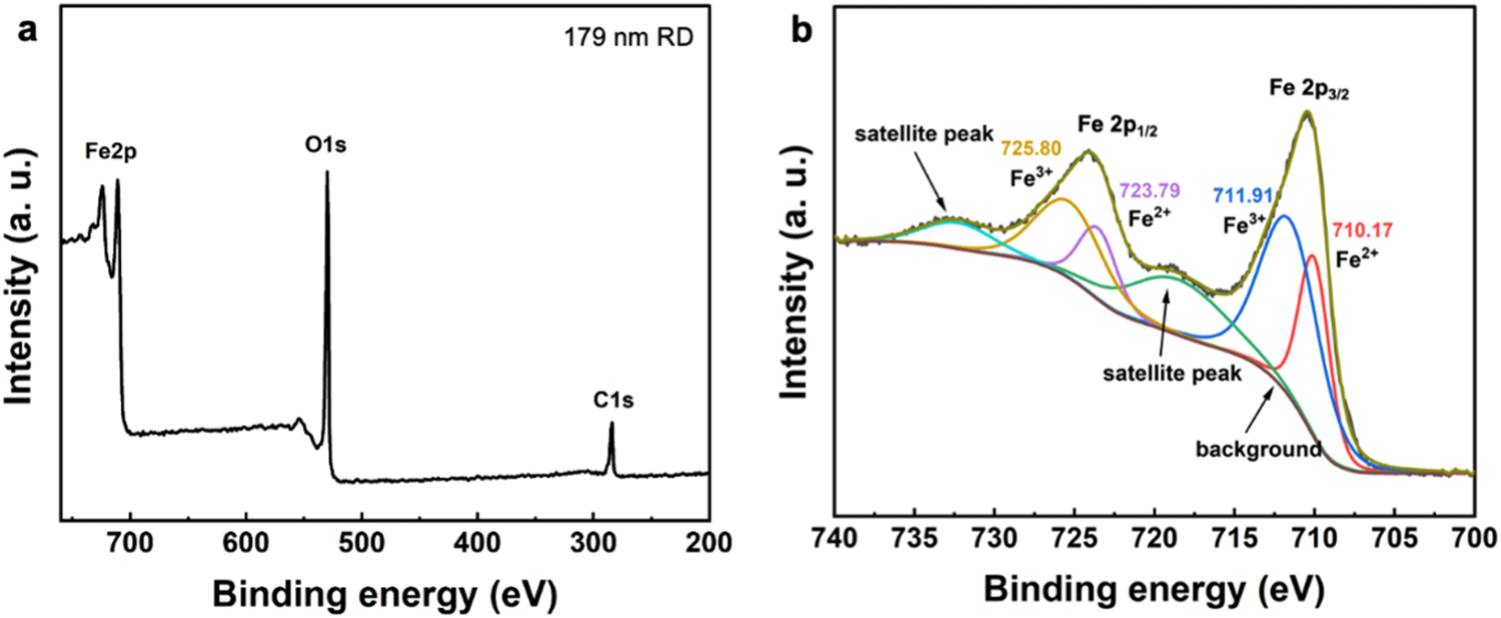
(a) Full XPS data of the 179 nm Fe_3_O_4_ rhombic dodecahedra. (b) Expanded spectrum showing the Fe peak region.

X-ray photoelectron spectroscopy was also employed to check if there are oxidation state and bonding environment differences in the synthesized Fe_3_O_4_ crystals. Figures 6 and S4 give the collected XPS results of the five samples. Both Fe and O signals are observed. The Fe 2p_1/2_ and Fe 2p_3/2_ peaks are well separated and can be deconvoluted into contributions from the Fe^3+^ and Fe^2+^ ions. For 179 nm rhombic dodecahedra, the Fe^3+^ peaks are at 725.8 and 711.9 eV, while Fe^2+^ peaks are located at approximately 723.8 and 710.2 eV. These iron peak positions match with literature values for Fe_3_O_4_.^28^ The satellite peaks are primarily due to multiplet splitting of the Fe^2+^ to Fe^3+^ ions and strong correlation effects, resulting in peaks at binding energies higher than those of the main Fe peaks. Remarkably, while XPS peak positions for truncated cubes and truncated rhombic dodecahedra are essentially identical, they differ noticeably from those of rhombic dodecahedra. The results support that surface lattice variations exist among the different particle morphologies.

### Optical and Magnetic Property Characterization

**Figure 7:**
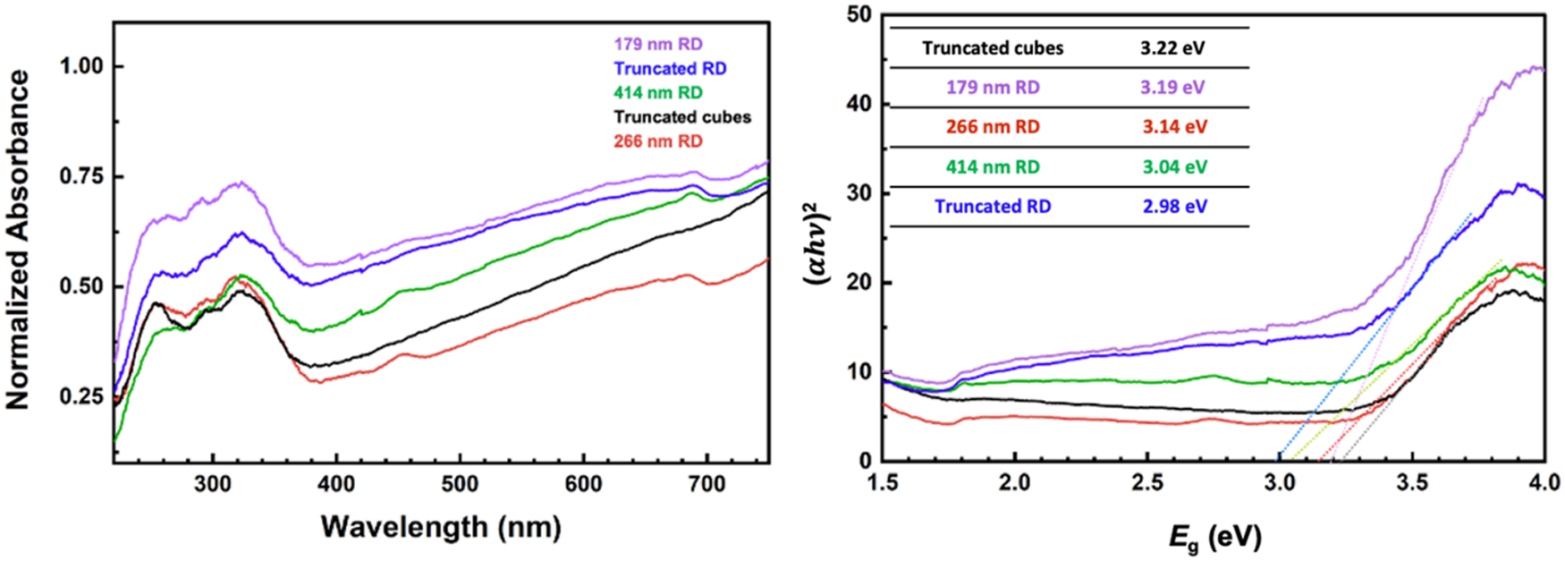
Diffuse reflectance spectra of the Fe_3_O_4_ crystals and the Tauc plot.

Diffuse reflectance spectra and the Tauc plot of all of the samples are provided in Figure 7. Fe_3_O_4_ nanoparticles are known to have band gaps of ∼2.1 and 3.3 eV.^29^ The larger band gap comes from valence band O^2–^ → conduction band Fe^3+^ charge transfer, while the O^2–^ →Fe^2+^ charge transfer gives the smaller band gap.^29^*d*-*d* intervalence charge transfer transitions, in which a d electron in a cation is transferred to a neighboring cation through optical excitation, have also been reported.^30^ Here, only one band gap can be clearly identified. For rhombic dodecahedra, band gap decreases with increasing particle size from 3.19 to 3.04 eV. This is the generally observed phenomenon, that semiconductor band gaps continue to change even for large nanocrystals.^31,32^ To demonstrate the presence of an optical facet effect, particle volumes need to be determined (Table S2). The {100}-truncated rhombic dodecahedra have the largest volume, while {110}-truncated cubes have a larger volume (1.57 × 10^7^ nm^3^) than that of 266 nm rhombic dodecahedra (1.12 × 10^7^ nm^3^). Figure S5 is a plot of the particle volumes versus their band gaps. The larger cubes actually have a band gap (3.22 eV) that is larger than that of the 266 nm rhombic dodecahedra (3.14 eV). This demonstrates the existence of optical facet dependence, in that exposure of {100} surfaces can absorb shorter light wavelengths.

**Figure 8:**
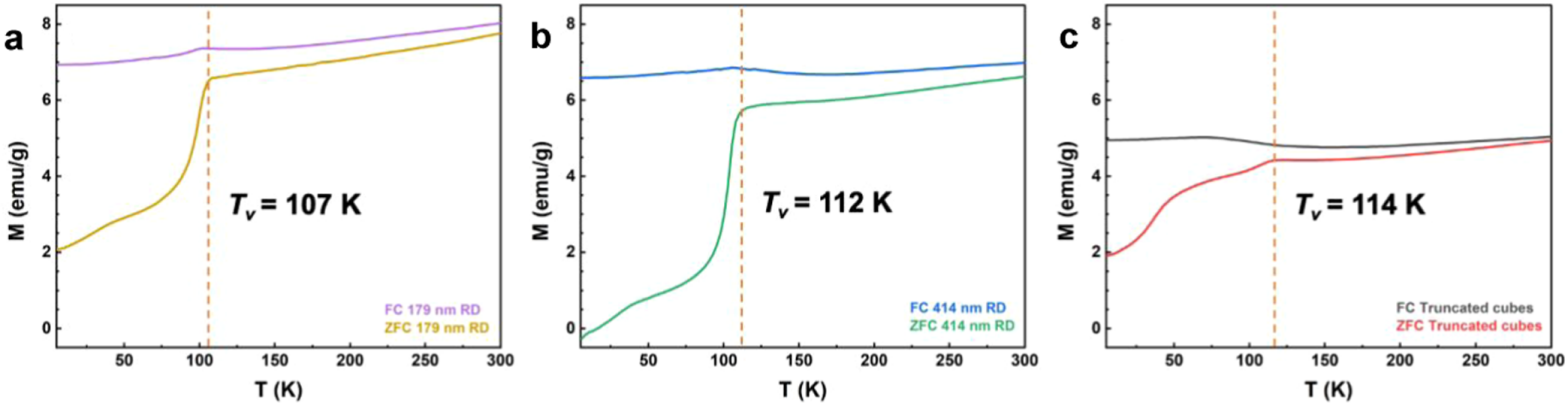
FC and ZFC curves measured at 300 K for Fe_3_O_4_: (a) 179 nm rhombic dodecahedra, (b) 414 nm rhombic dodecahedra, and (c) truncated cubes.

For magnetic property examination, zero-field-cooling (ZFC) and field-cooling (FC) magnetization measurements were performed from 5 to 300 K under an applied magnetic field of 100 Oe at 300 K (Figure 8). Under ZFC condition, the 414 nm rhombic dodecahedra are only weakly magnetized at low temperatures. It then increases steeply up to 5.72 emu/g reaching the Verwey transition (*T*_V_) at 112 K. For the truncated cubes and 179 nm rhombic dodecahedra, they show a higher magnetization value of ∼2 emu/g at 5 K and respective magnetization transition temperatures at 114 and 107 K. Above the Verwey temperature, the magnetization gradually increases up to 300 K. The Verwey transition is a structural transformation that can occur at about 125 K for Fe_3_O_4_.^33^ Above the Verwey transition, Fe_3_O_4_ is a conductive metal oxide with an inverse cubic spinel structure. However, upon cooling Fe_3_O_4_ below *T*_v_, it becomes less conductive or an insulator and adopts a distorted monoclinic crystal structure. The abrupt change in crystallographic structure is accompanied by changes in magnetic, electric, thermodynamic, and mechanical properties. For the FC measurements, the magnetization slightly increases and then decreases slowly around the Verway transition. The *M-T* traces for rhombic dodecahedra are more similar, while edge-truncated cubes exhibit more distinct traces. It is suggested that the lattice variations are linked to this phenomenon. The FC and ZFC curves converge at high temperatures, meaning that the blocking temperature, at which the exchange field becomes zero, is beyond 300 K for all of the Fe_3_O_4_ samples.

**Figure 9:**
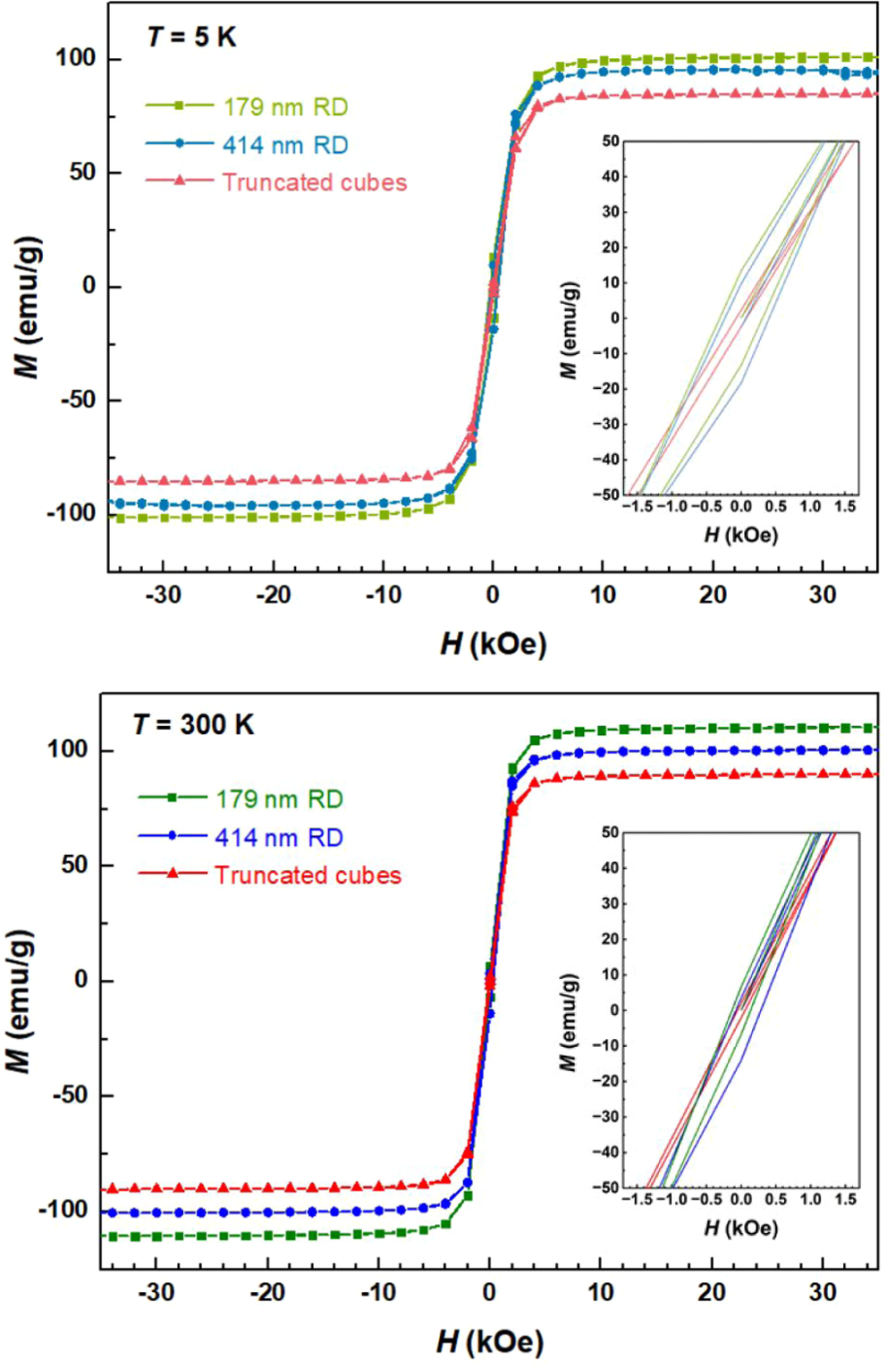
*M-H* curves for different Fe_3_O_4_ samples measured at 5 and 300 K.

The magnetization versus applied magnetic field curves (*M-H* curves) for the three samples measured at 5 and 300 K are provided in Figure 9. The expanded *M-H* curves of these Fe_3_O_4_ crystals are available in Figure S6. All samples exhibit ferrimagnetic behavior with coercivity (*H*_c_) and remanence (*M*_r_). First, all the samples have generally large saturation magnetization (*M*_s_), coercivity, and remanence values. Table 1 summarizes the values for the magnetic parameters. Unexpectedly, the samples actually have higher magnetization values at room temperature than at 5 K. The smallest 179 nm rhombic dodecahedra have the largest *M*_s_ value of 110.63 emu/g at 300 K, while 414 nm rhombic dodecahedra and truncated cubes have recorded *M*_s_ values of 100.30 and 90.02 emu/g, respectively. These numbers are appreciably larger than *M*_s_ values reported for diverse Fe_3_O_4_ nanostructures including cubes, octahedra, rhombic dodecahedra, and spheres.^15,18,34−38^ For example, 287 nm cube-like Fe_3_O_4_ particles have a *M*_s_ value of 84.7 emu/g, and 87.5 and ∼80 emu/g for 500 nm octahedra and 500 nm rhombic dodecahedra, respectively.^15,18,34^ The obtained coercivity values (302.4 and 137.5 Oe for 179 nm rhombic dodecahedra at 5 and 300 K) are also higher compared to many Fe_3_O_4_ reports with numbers below 100 Oe. While saturation magnetization is normally higher for bigger Fe_3_O_4_ particles, there are also cases where smaller particles have larger *M*_s_ values.^15,38^ Instead of considering surface magnetic anisotropy from the coordination environment of surface atoms, the recorded facet- and size-related magnetic responses can similarly be rationalized recognizing that there are lattice variations among these three samples, and 179 nm rhombic dodecahedra have a particularly thick surface layer. It is the sample-specific lattice deviations in the crystal interior that lead to these magnetic behaviors. Thus, by making these rhombic dodecahedra, particles with stronger magnetic responses can be attained for applications such as protein detection and magnetic resonance imaging.^39^

**Table 1:** Magnetic Parameters of Different Fe_3_O_4_ Crystals

	5 K			300 K		
	*M*_S_ (emu/g)	*M*_r_ (emu/g)	*H*_C_ (Oe)	*M*_S_ (emu/g)	*M*_r_ (emu/g)	*H*_C_ (Oe)
truncated cubes	85.09	2.35	72.16	90.02	2.08	55.13
414 nm RD	95.43	9.78	237.18	100.30	3.54	77.24
179 nm RD	101.13	13.08	302.44	110.63	6.77	137.49

Moreover, the superparamagnetic behavior has been reported to occur in Fe_3_O_4_ crystals containing <15 nm crystallites.^37^Figure 5 indicates that the entire particle is a single crystal, so the observed magnetic properties should not be attributed to superparamagnetism. Any change in magnetic dipole direction cannot be easily probed since the huge number of nanoparticles loaded on a surface can have all orientations and can stack significantly from their magnetic interactions. Furthermore, given the very small unit cell constant changes on the order of 0.01–0.02 Å among these samples, as seen in Table S1, it is likely that the antiferrimagnetic superexchange interactions between the crossed Fe_oct_^3+^ and Fe_tet_^3+^ ions through the bridging O^2–^ anions, and ferrimagnetic double-exchange interactions between adjacent Fe_oct_^3+^ and Fe_oct_^2+^ ions, should remain the same for these Fe_3_O_4_ crystals.^40,41^

## Conclusions

Fe_3_O_4_ edge-truncated cubes, corner-truncated rhombic dodecahedra, and size-tunable rhombic dodecahedra were solvothermally synthesized. In addition to in-house XRD patterns, synchrotron XRD analysis reveals the presence of bulk and surface layer lattice components, with the smallest 179 nm rhombic dodecahedra having a substantially thick surface layer. TEM characterization shows large lattice deviations in the surface layer region. XPS results indicate Fe peak shifts between truncate cubes and rhombic dodecahedra. Both size- and facet-dependent band gap changes have been identified. While all of the measured samples have relatively large saturation magnetization values, 179 nm rhombic dodecahedra have the largest *M*_s_, *M*_r_, and *H*_c_ values, followed by 414 nm rhombic dodecahedra and then truncated cubes. Both the optical and magnetic behaviors can be linked to their lattice variations. Thus, it is necessary to examine the entire crystal interior to understand the shape effect on magnetic responses.

## Experimental Section

### Chemicals

Ferrous sulfate, 7-hydrate (J. T. Baker), lithium nitrate (99%, Alfa Aesar), urea (CH_4_N_2_O, 99.0–100.5%, J. T. Baker), hydrogen peroxide (35%, Showa), 1-butanol (C_4_H_9_OH, 99.4%, Thermal), and toluene (C_6_H_5_CH_3_, > 99.5%, J. T. Baker) were used.

### Synthesis of Fe_3_O_4_ Edge-Truncated Cubes and Truncated Rhombic Dodecahedra

Glass tubes were used for the growth of Fe_3_O_4_ edge-truncated cubes and truncated rhombic dodecahedra. Deionized water and 1-butanol were first placed in glass tubes. Varying volumes of 1 M lithium nitrate solution, 2 mL of 0.05 M FeSO_4_ solution, and 4.5 mL of 2 M urea solution were quickly introduced. The total solution volume is 20 mL. The reaction mixture was then sonicated at room temperature for 15 min, and the sealed tubes were transferred to an oven and heated to 160 °C for 2 h to make truncated cubes and 30 min for truncated rhombic dodecahedra. See Table S3 for the reagent amounts used. After the reaction, the particles were cooled to room temperature and separated using centrifugation at 10000 rpm for 10 min. The product was washed three times with a 1:1 volume ratio of 95% ethanol and deionized water.

### Synthesis of Size-Tunable Fe_3_O_4_ Rhombic Dodecahedra

For the growth of size-tunable Fe_3_O_4_ rhombic dodecahedra, 3.525 mL of deionized water, 3.525 mL of toluene, specified amounts of hydrogen peroxide, and 0.2 mL of 1 M lithium nitrate were mixed in a vial. By gradual increase of the amount of hydrogen peroxide, it is possible to produce smaller rhombic dodecahedra. Next, 1 mL of 0.05 M FeSO_4_ solution and 1.75 mL of 2 M urea were added with stirring for 15 min. The total solution volume is 10 mL. Table S4 lists the reagent amounts used for the particle size variation. Use of a glass tube to synthesize rhombic dodecahedra was found to give particle size inhomogeneity. The solution was then loaded into a Teflon-lined stainless-steel autoclave, transferred to an oven, and heated at 180 °C for 2 h. The black product was transferred to centrifuge tubes and rinsed 3 times with a 1:1 volume ratio of 95% ethanol and deionized water. The setting of the high-speed centrifuge was 10,000 rpm for 5 min each time. The pure product was vacuum-dried and stored at room temperature.

### Synchrotron XRD Measurements and Analysis

High-resolution powder X-ray diffraction data were collected at the Taiwan Photon Source 19A beamline (TPS19A) at the National Synchrotron Radiation Research Center (NSRRC), Taiwan. The 22.13 keV hard X-ray source (0.56023 Å wavelength) was generated by an in-vacuum cryogenic undulator (CU15), and diffraction patterns were captured using a position-sensitive detector, MYTHEN 18K. For high-resolution powder diffraction analysis, a unique axis microstrain model was incorporated into the Rietveld refinement, which was performed using GSAS-II software. This model accounted for the exposed facets of the nanocrystals with the direction of each facet defined as the unique axial direction.

### Instrumentation

For XRD measurements, a Bruker D2 PHASER diffractometer operating at 30 kV and 10 mA with Cu *K*α radiation was applied. For SEM images, a JEOL JSM-7000F electron microscope was used. TEM images were obtained using a spherical aperture-corrected emission transmission electron microscope (JEOL, JEM-ARM200FTH). Surface analysis was conducted with a high-resolution ULVAC-PHI PHI Quantera II X-ray photoelectron spectrometer. Ultraviolet–visible absorption spectra were collected using a JASCO V-670 spectrometer with diffuse reflectance spectroscopy mode. A Quantum Design MPMS-3 superconducting quantum interference device magnetometer (SQUID) was applied for magnetic measurements.

## Supporting Information Available

Particle size distribution histograms; XRD patterns; Rietveld refinement results; XPS spectra; particle volume to band gap plot; expanded *M-H* curves, and particle synthesis conditions (PDF)
