# Supplementary material for: Growth of Fe3O4 Truncated Cubes and Rhombic Dodecahedra Showing Interior Lattice and Magnetic Behavior Variations
Source: Inorg Chem. 2025 Apr 23;64(17):8659–67. doi: 10.1021/acs.inorgchem.5c00442 (PMC12056693; doi:10.1021/acs.inorgchem.5c00442)
Supplement: Supplementary file 1 — ic5c00442_si_001.pdf [file ic5c00442_si_001.pdf]

Supporting Information

Growth of Fe_3O_4 Truncated Cubes and Rhombic Dodecahedra Showing Interior Lattice and Magnetic Behavior Variations

Jou-Hsin Yang,[†] Chia-Peng Wang,[†] Bo-Hao Chen,[†‡] and Michael H. Huang*[†]

[†]*Department of Chemistry, National Tsing Hua University, Hsinchu 300044, Taiwan*

[‡]*National Synchrotron Radiation Research Center, Hsinchu 300092, Taiwan*

Email: hyhuang@mx.nthu.edu.tw

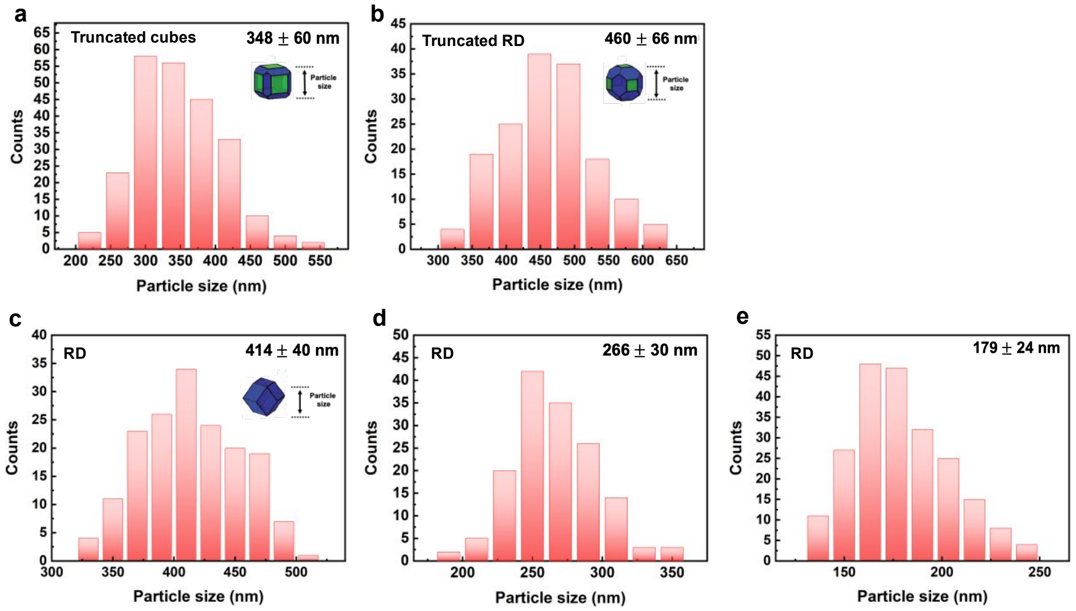

Figure S1. Size distribution histograms of the synthesized Fe_3O_4 (a) truncated cubes, (b) truncated rhombic dodecahedra, (c) 414 nm rhombic dodecahedra, (d) 266 nm rhombic dodecahedra, and (e) 179 nm rhombic dodecahedra.

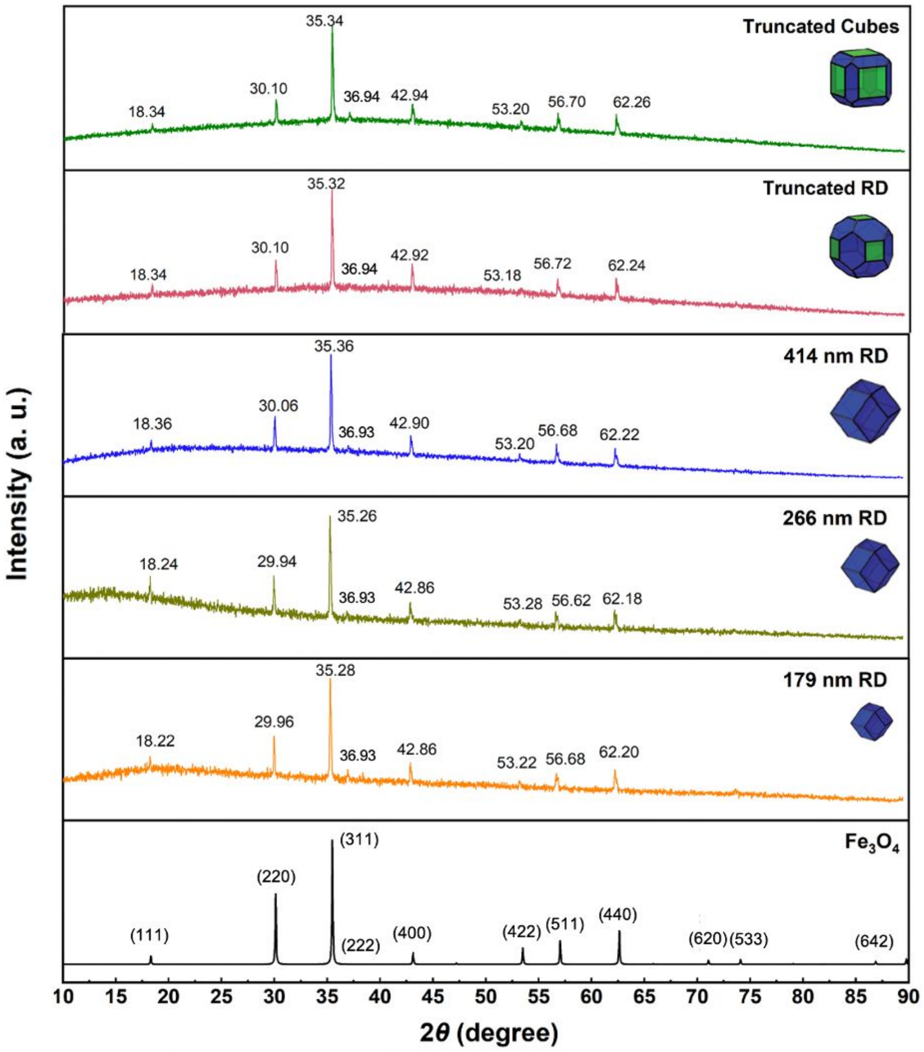

Figure S2. XRD patterns of the prepared Fe_3O_4 crystals and a reference pattern.

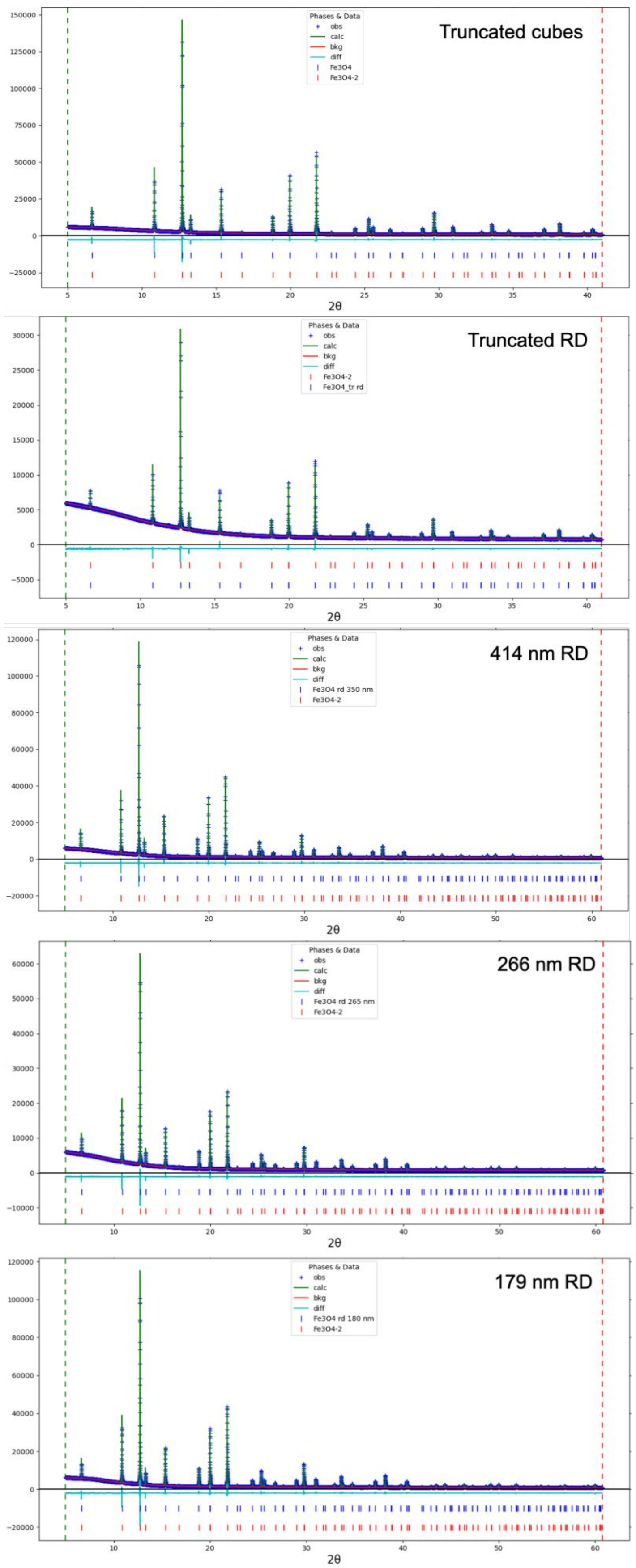

Figure S3. Rietveld refinement of different Fe_3O_4 crystals.

Table S1. Lattice parameters of Fe_3O_4 crystals after Rietveld refinement of synchrotron XRD patterns.

Morphology	Truncated Cubes		Truncated Rhombic Dodecahedra		Rhombic Dodecahedra 414 nm		Rhombic Dodecahedra 266 nm		Rhombic Dodecahedra 179 nm	
	Bulk	Surface	Bulk	Surface	Bulk	Surface	Bulk	Surface	Bulk	Surface
Unit cell a (Å)	8.40167(2)	8.3932(4)	8.40177(3)	8.39615(23)	8.39518(2)	8.38425(8)	8.39500(2)	8.38452(12)	8.38732(3)	8.37764(10)
Weight percentage (%)	85.8(6)	14.2(6)	81.5(9)	18.5(9)	84.4(2)	15.6(2)	77.8(4)	22.2(4)	53.1(4)	46.9(4)
Equatorial μ strain $\Delta d/d$ ($\times 10^{-6}$)	2573	5331	Microstrain 2513	3774	2480	3054	3048.9	3599.0	3408	4959
Axial μ strain $\Delta d/d$ ($\times 10^{-6}$)	2674	4231		3852	2265	3129	2732.4	3985.6	2652	5014
μ strain unique axis (hkl)	100		isotropic	110	110		110		110	
Background function	log interpolate		log interpolate		log interpolate		log interpolate		log interpolate	
2 theta range (degree)	5.0 ~ 61.0		5.0 ~ 41.0		5.0 ~ 61.0		5.0 ~ 61.0		5.0 ~ 60.75	
d-spacing resolution (Å)	0.556		0.808		0.555		0.555		0.555	
Zero-point shift (degree)	-0.0001		-0.0006		-0.0005		-0.0016		-0.0003	
wR (%)	4.09		1.79		3.44		2.87		4.61	
R (F^2) (%)	4.53	5.31	7.89	9.73	6.66	4.46	4.86	3.64	5.46	3.78

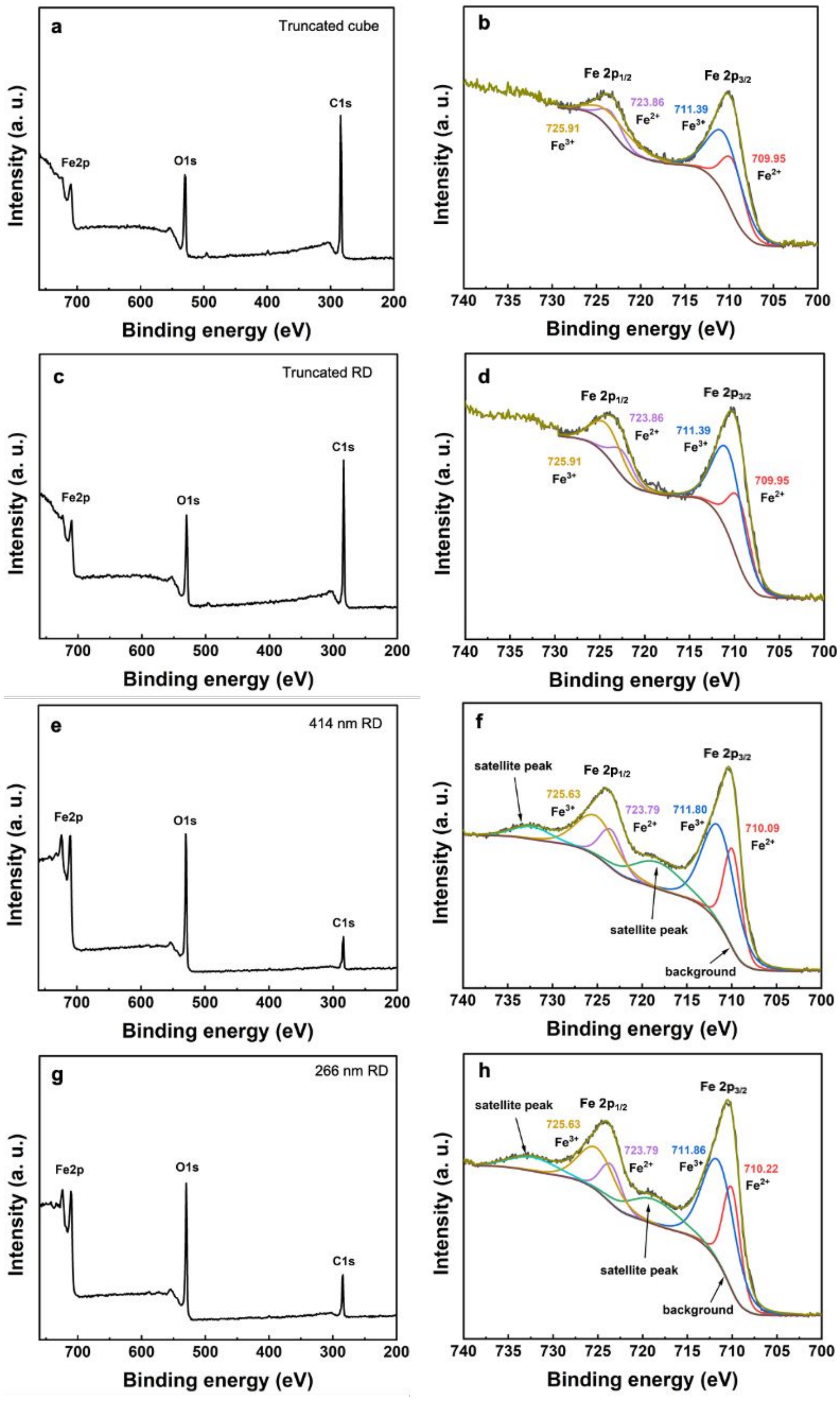

Figure S4. Full and expanded XPS data of Fe_3O_4 (a, b) truncated cubes, (c, d) truncated rhombic dodecahedra, (e, f) 414 nm rhombic dodecahedra, and (g, h) 266 nm rhombic dodecahedra.

Table S2. Calculations of the particle volumes.

	Truncated cubes	Truncated RD	RD		
Particle size	348 nm	460 nm	414 nm	266 nm	179 nm
	$V = b^3 - (b^3 - a^3)$ 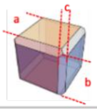	$V = b^3 - (b^3 - a^3)$ 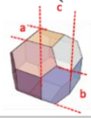	$V = \frac{16}{9} \times a^3 \times \sqrt{3}$ 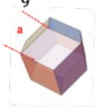		
	a = 250 nm<br>b = 303 nm<br>c = 65 nm	a = 152 nm<br>b = 309 nm<br>c = 214 nm	a = 239 nm	a = 154 nm	= 103 nm
Volume per particle (nm^3)	1.57×10^7	5.54×10^7	4.20×10^7	1.12×10^7	3.40×10^6

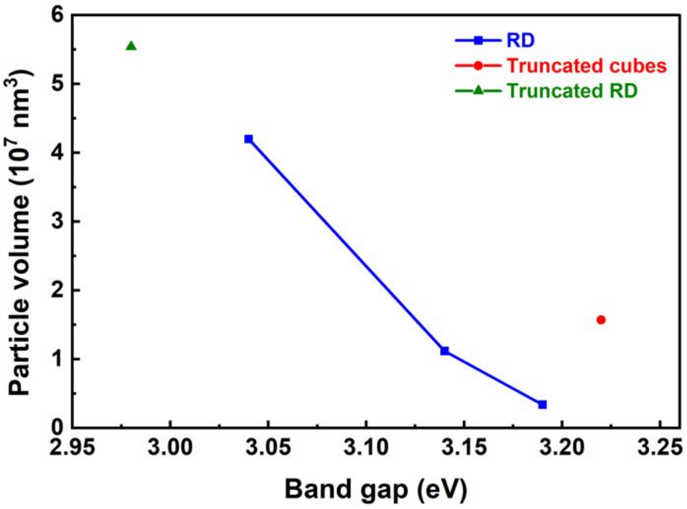

Figure S5. Plot of particle volumes versus their band gaps.

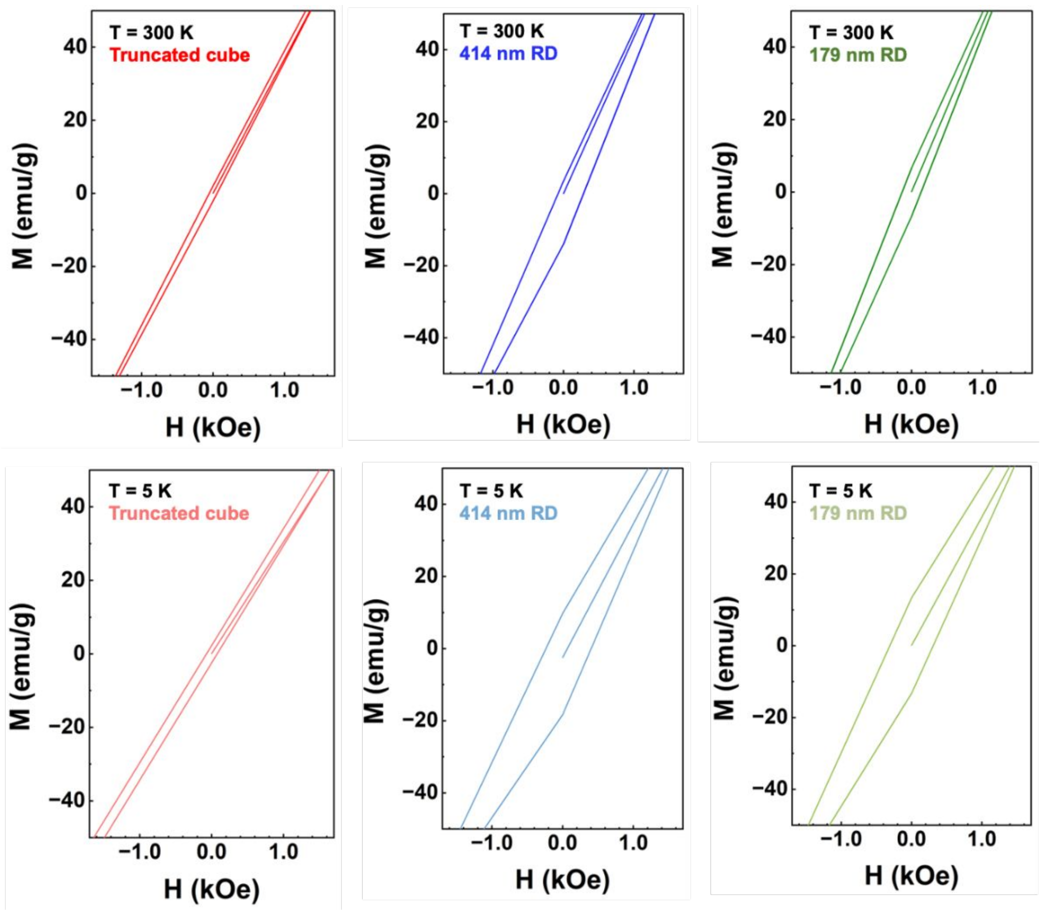

Figure S6. Expanded *M-H* curves of the Fe_3O_4 crystals.

Table S3. Reagent amounts used for the synthesis of Fe_3O_4 truncated cubes and truncated rhombic dodecahedra

	1-butanol	$LiNO_3$ (1 M)	$FeSO_4$ (0.05 M)	urea (2 M)	total volume	temp.	time
**348 nm truncated cubes**	1.0 mL	0.7 mL	2.0 mL	4.5 mL	20 mL	160 °C	2 h
**460 nm truncated RD**	3.0 mL	2.5 mL	2.0 mL	4.5 mL	20 mL	160 °C	0.5 h

Table S4. Reagent amounts used for the growth of size-tunable Fe_3O_4 rhombic dodecahedra

	toluene	H_2O_2	$LiNO_3$ (1 M)	$FeSO_4$ (0.05 M)	urea (2 M)	H_2O	temp.	time
**414 nm RD**	3.525 mL	2.5 μL	0.2 mL	1.0 mL	1.75 mL	3.525 mL	180 °C	2 h
**266 nm RD**	3.525 mL	8 μL	0.2 mL	1.0 mL	1.75 mL	3.525 mL	180 °C	2 h
**179 nm RD**	3.525 mL	30 μL	0.2 mL	1.0 mL	1.75 mL	3.525 mL	180 °C	2 h
